# Concentration Dependent Ion Selectivity in VDAC: A Molecular Dynamics Simulation Study

**DOI:** 10.1371/journal.pone.0027994

**Published:** 2011-12-02

**Authors:** Eva-Maria Krammer, Fabrice Homblé, Martine Prévost

**Affiliations:** Structure et Fonction des Membranes Biologiques, Université Libre de Bruxelles, Brussels, Belgium; University of Rome, Italy

## Abstract

The voltage-dependent anion channel (VDAC) forms the major pore in the outer mitochondrial membrane. Its high conducting open state features a moderate anion selectivity. There is some evidence indicating that the electrophysiological properties of VDAC vary with the salt concentration. Using a theoretical approach the molecular basis for this concentration dependence was investigated. Molecular dynamics simulations and continuum electrostatic calculations performed on the mouse VDAC1 isoform clearly demonstrate that the distribution of fixed charges in the channel creates an electric field, which determines the anion preference of VDAC at low salt concentration. Increasing the salt concentration in the bulk results in a higher concentration of ions in the VDAC wide pore. This event induces a large electrostatic screening of the charged residues promoting a less anion selective channel. Residues that are responsible for the electrostatic pattern of the channel were identified using the molecular dynamics trajectories. Some of these residues are found to be conserved suggesting that ion permeation between different VDAC species occurs through a common mechanism. This inference is buttressed by electrophysiological experiments performed on bean VDAC32 protein akin to mouse VDAC.

## Introduction

The voltage-dependent anion channel (VDAC) is the most abundant integral membrane protein of the mitochondrial outer membrane. It is a key regulator of metabolite flow notably of adenosine nucleotides, sugars and inorganic ions and forms the main interface between the mitochondrial and the cytoplasmic metabolism [1]. Several studies also point to an involvement of VDAC in various cell processes including apoptosis, calcium homeostasis and diseases such as cancer ([2] and references therein).

Many different organisms possess multiple VDAC isoforms though their number varies depending on the species [2]–[6]. All however appear to have at least one isoform that features canonical electrophysiological properties particularly important for the transport of metabolites across the mitochondrial outer membrane [7]–[9]. The physiological significance of this functionally conserved VDAC isoform is thought to be strongly correlated to its voltage-dependence [10]. At voltages close to 0 mV, the channel exists in a fully open state characterized by a high conductance of about 4 nS (in 1.0 M KCl) [7], [8], which is compatible with the magnitude of the metabolites flow into and out of the mitochondria. Upon higher voltages (>±20 mV) VDAC switches to partially closed states showing lower conductances for small ions and being no longer permeable to metabolites. In its open state, this channel possesses a slight preference for inorganic anions over cations while it shows a reversed selectivity in most of its closed states [1], [7], [8], [11]. There is also some evidence that the electrophysiological properties (conductance, reversal potential) vary with the bulk salt concentration [7]–[9], [12]–[16].

**Figure 1:**
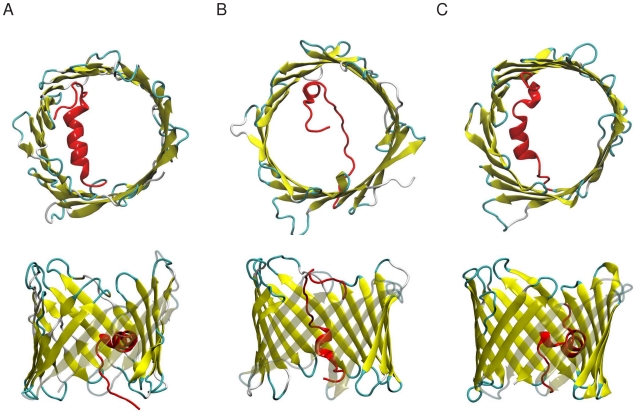
The structure of VDAC. Top and side view of the three atomic resolution structures determined for (A) mVDAC1 and (B,C) hVDAC1 determined by either Xray crystallography (A; pdb code 3emn), by NMR (B; pdb code 2k4t), or by a combination of both methods (C; pdb code 2jk4) [17]–[19]. These structures mainly differ in the position of the N-terminal segment (highlighted in red). The protein structures are depicted as cartoon. Only the best representative conformer is shown for the NMR structure. All images were prepared with vmd [76].

Three experimental three-dimensional (3D) structures of mouse (mVDAC1) and human VDAC1 isoform (hVDAC1) have been recently determined by X-ray crystallography and NMR (Figure 1). These structures have dramatically changed the molecular picture of the channel [17]–[19]. They reveal a pore-forming protein shaped as a β-barrel comprising 19 antiparallel β-strands closed by two parallel strands. Because of the unusual odd number of β-strands compared to that observed in the structures of bacterial β-barrel proteins [3], [20] and because of conflicts with biochemical and functional data the biological significance of these atomic resolution structures has been questioned [21]. However, the atomic resolution structures and additional NMR measurements of VDAC1 in detergent micelles and DMPC nanodiscs share all a remarkable similar structure although the data were obtained in different detergent and lipid environments [22], [23]. Moreover, spectroscopic and bioinformatic studies corroborate that VDAC channels purified from fungi, plants and mammals have similar secondary structure content and topology coherent with the atomic resolution structures [9], [24]–[28]. Furthermore, theoretical studies based on the NMR hVDAC1 and crystal mVDAC1 concluded that these pores are anion-selective [29]–[31] sustaining the biological relevance of these 3D structures.

In contrast to earlier structural models [21], [32], [33], in which the N-terminal region (residues 1 to 20) was suggested to be part of the barrel or to lie outside the barrel, all three 3D structures agree on the location of the N-terminal segment inside the pore. They differ however on the exact position of this segment, on its local structure and on its interactions with the barrel (Figure 1). In the crystal mVDAC1 structure and the mixed Xray/NMR hVDAC1 the N-terminal segment adopts an almost full α-helix that is nearly perpendicular to the β-barrel axis and that leans onto the barrel wall at about the middle of the pore thereby restricting its size. The NMR conformations of hVDAC1 feature a shorter helix (residues 6 to 11) and a rather undefined structure for the remaining residues, hinting at the flexibility of this segment. A recent solid-state NMR experiment [34] on hVDAC1 sustained that the N-terminus adopts a well-defined helical conformation as in the mVDAC1 crystal and the hVDAC1 mixed X-ray/NMR structures [18], [19]. To explain these structural discrepancies it was proposed that the hVDAC1 NMR conformations describe (partially) closed states while the mVDAC1 crystal structure depicts the fully open state [35]. Nonetheless, recent theoretical studies concluded that the hVDAC1 NMR structures are anion-selective states and might represent open states [30], [31]. Thus, whether the N-terminal region owns an intrinsic flexibility and whether this flexibility is of biological relevance remain elusive so far.

Albeit VDAC has been extensively studied to understand its ion transport properties there is still a number of issues that need to be addressed. The combination of now available atomic resolution structures with computer modeling and simulation tools gives the possibility of unraveling, at the molecular level, the fundamental principles of ion translocation through the channel. Continuum electrostatic calculations and Brownian dynamics (BD) simulations were recently carried out to get insight into the anion-selectivity of mVDAC1 [29] and hVDAC1 [31]. Detailed all-atom molecular dynamics (MD) simulations accounting for the fluctuations of the protein structure and describing explicitly the protein surroundings were performed to study the importance of the residue E73 for the dynamics of mVDAC1 [36] and to investigate the electrophysiological properties of the hVDAC1 structure [30]. An aspect which has so far not been studied is the dependence of the ion permeation upon salt concentration. We investigated, at the atomic detail, the ion translocation process at different salt concentrations using a combination of MD and continuum electrostatic approaches carried out on the crystal structure of mVDAC1. Our MD simulations were performed at KCl concentrations ranging from 0.1 M to 1.4 M and analyzed to understand the molecular mechanism of ion crossing through the channel. In order to identify possible conserved mechanistic elements of VDAC selectivity an aminoacid conservation study was performed and electrophysiological experiments were carried out on VDAC32 from the bean *Phaseolus sp.* which was reported to feature electrophysiological properties similar to mVDAC1 [9], [37]. This work contributes to provide new insight into the molecular determinants of ion transport in VDAC.

## Results

### Structural integrity of mVDAC1 is kept during the MD simulations

Two 50 ns MD simulations of mVDAC1 in an explicit lipid bilayer were carried out at two different ionic concentrations: 0.1 M and 1.0 M KCl. These concentrations were chosen since they are either representative of physiological conditions (0.1 M KCl) [38] or are routinely used in experiments (1.0 M KCl).

To assess the stability of mVDAC1 in our MD simulations, we computed the root-mean-square deviation (RMSD) of the backbone and side chain heavy atom positions using the crystal structure as a reference. In all four 50 ns trajectories the backbone and side chain RMSD values are on average inferior to 2 Å indicating that the protein remains stable during the simulations (Table 1). The flexibility of the protein was also analyzed by calculating the root mean-square fluctuations (RMSF) of each residue from its time-average position. The RMSFs are similar in all four simulations (Figure S1). Qualitatively they also agree well with the B factors of the mVDAC1 crystal structure [19] reproducing the alternation between the more rigid β-strand segments and the more flexible loops.

**Table 1:** Structural, dynamic and ion-channel properties computed from the MD simulations.

[KCl]	Quantity	Set-up 1	Set-up 2
0.1 M	backbone RMSD	1.6±0.2 Å	1.5±0.2 Å
	sidechain RMSD	2.6±0.1 Å	2.6±0.1 Å
	time-averaged number of Cl^−^ inside the pore	1.8±0.9	2.4±1.0
	time-averaged number of K^+^ inside the pore	0.3±0.5	0.2±0.4
	translocation events for Cl^−^/K^+^	14/1	13/1
	average passage time for Cl^−^	2.5±1.8 ns	2.7±1.5 ns
	average passage time for K^+^	-	-
	N_Cl−_/N_K+_ ratio	7.0	13.1
	permeation ratio	14.0	13.0
1.0 M	backbone RMSD	1.8±0.1 Å	1.6±0.1 Å
	sidechain RMSD	2.8±0.1 Å	2.7±0.1 Å
	time-averaged number of Cl^−^ inside the pore	11.3±2.2	10.6±2.4
	time-averaged number of K^+^ inside the pore	7.6±2.2	7.3±2.3
	translocation events for Cl^−^/K^+^	82/51	67/40
	average passage time for Cl^−^	2.1±1.4 ns	2.1±1.8 ns
	average passage time for K^+^	1.9±1.3 ns	1.8±1.1 ns
	N_Cl−_/N_K+_ ratio	1.49	1.45
	permeation ratio	1.61	1.67

The time-averaged backbone RMSD (relative to the crystal structure), the time-averaged side chain RMSD, the number of ions inside the pore (N_Cl_− and N_K+_), the number of translocation events and the average passage time computed using the 0.1 and 1 M KCl simulations are listed. In addition to N_Cl_−/N_K+_ ratio, the ratio between the number of translocation events for chloride and potassium (permeation ratio) was computed. A translocation event was defined as the event of an ion traveling across the pore axis from z<15 to z>−15 or vice versa. The pore was defined as −15<z<15 Å. The average translocation time for potassium could not be computed at 0.1 M since only a single translocation event occurred. The side chain RMSD was calculated for heavy atoms only excluding Lys161, since its side chain position was not determined in the crystal structure (pdb 3emn; [19]).

### mVDAC1 shows a preference for anions

**Figure 2:**
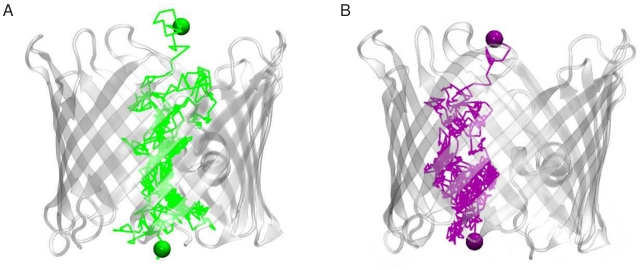
Translocation of ions through VDAC. The translocation path of (A) a Cl^−^ (in green) and (B) a K^+^ (in purple) ion through mVDAC1 was extracted from the first 0.1 M KCl simulation. The start and end positions of the permeating Cl^−^/K^+^ ion are highlighted as a green/purple sphere. The broken lines connect the positions of the chloride or potassium ion separated by a timestep of 4 ps. The protein is shown as a transparent-white cartoon.

Our MD trajectories at 0.1 M and 1.0 M KCl were analyzed to examine the dynamics of the ions inside the pore. In all four simulations performed under equilibrium conditions spontaneous crossings of chloride and potassium ions through the pore were observed irrespective of the bulk salt concentration (Figure 2, Figure S2). We thus computed the time-averaged number of K^+^ (N_K+_) and of Cl^−^ (N_Cl−_) visiting the pore and the number of translocation events of K^+^ and of Cl^−^ through the channel. As shown in Table 1, the two quantities are always higher for chloride than for potassium indicating a preference of the pore for chloride. No significant difference is observed in the translocation times between potassium and chloride (Table 1).

### Distribution of anions and cations in the pore depends on the solution concentration

To study the concentration dependence of the chloride and potassium permeation across VDAC [14], [39], additional MD simulations were carried out at KCl concentration values ranging from 0.2 M to 1.4 M KCl.

**Figure 3:**
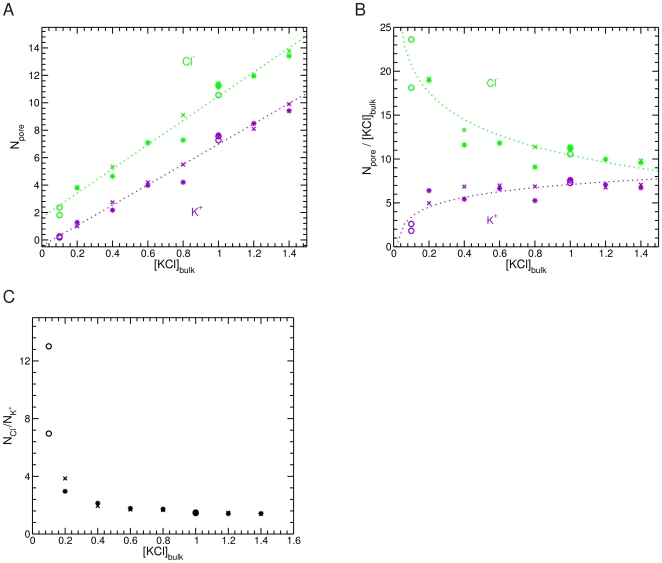
The ion distribution inside mVDAC1 depends on the bulk KCl concentration. (A) Number of Cl^−^ (green) and K^+^ (purple) ions inside the channel (N_pore_) versus the bulk concentration ([KCl]_bulk_). (B) Partition of Cl^−^ (green) and K^+^ (purple) ions in the pore relative to the bulk (N_pore_/[KCl]_bulk_) as a function of the bulk salt concentration [KCl]_bulk_. (C) N_Cl−_/N_K+_ ratio inside the pore as a function of [KCl]_bulk_. The data were extracted from two independent 15 ns simulations shown as crosses and stars, respectively at 0.2, 0.4, 0.6, 0.8, 1.0, 1.2 and 1.4 M KCl solution concentration and from two independent 50 ns simulations shown as circles at 0.1 and 1.0 M KCl solution concentration.

N_K+_ and N_Cl−_ are found to vary linearly with the salt concentration (Figure 3A). At all concentrations N_Cl−_ is larger than N_K+_. The partition of ions between the pore and the bulk varies in a non-linear manner with the concentration. For anions this partition dramatically decreases with increasing salt concentration while that of cations increases smoothly (Figure 3B). The ratio between N_Cl−_ and N_K+_ (N_Cl−_/N_K+_) drops significantly from 3.5 at 0.2 M KCl to 1.4 at 0.8 M KCl. From 0.8 M up to 1.4 M, the N_Cl−_/N_K+_ ratio inside the pore remains almost constant (Figure 3C). Adding more ions to the system does thus not lead to ionic saturation of the channel. These data are in agreement with experimental results showing that the anion selectivity decreases with increasing ionic strength [14].

### Molecular basis for the salt concentration effect

VDAC shares a similar architecture and function with the bacterial outer membrane porins. For instance, OmpF of *E. coli* is a voltage-dependent and cation-selective pore that is required for the transport of charged species across the outer membrane. Its selectivity and conductance were found to be concentration-dependent [40]–[48]. Several features of OmpF such as the existence of different pathways for potassium and chloride ions, of a transversal field in the constriction zone of the pore, partial dehydration of the ions, an increase in ion-pairing formation, especially in the constriction zone, and the major role of the protein electrostatic features were reported to explain the salt concentration dependence of its electrophysiological properties. We thus examined our MD trajectories at 0.1 M and 1.0 M KCl to analyze whether these properties can provide insight into the salt dependence of the ion permeation in VDAC.

#### Ion dehydration, long-lived protein-ion interactions and specific pathways are not observed during ion translocation

In our MD trajectories only a slight dehydration around the ions (less than one water molecule; Figure S3) is observed inside the pore irrespective of the KCl concentration. A dehydration of up to three water molecules in the first hydration layer was reported in OmpF [44].

Moreover, no long-lived (≥5 ns) interactions are formed between chloride or potassium and protein residues within the channel (Figure S4). A few short-lived ion-protein interactions are however identified, in particular for potassium at high KCl concentrations. Most of those interactions occur between potassium and negatively charged residues in loop regions of the protein.

**Figure 4:**
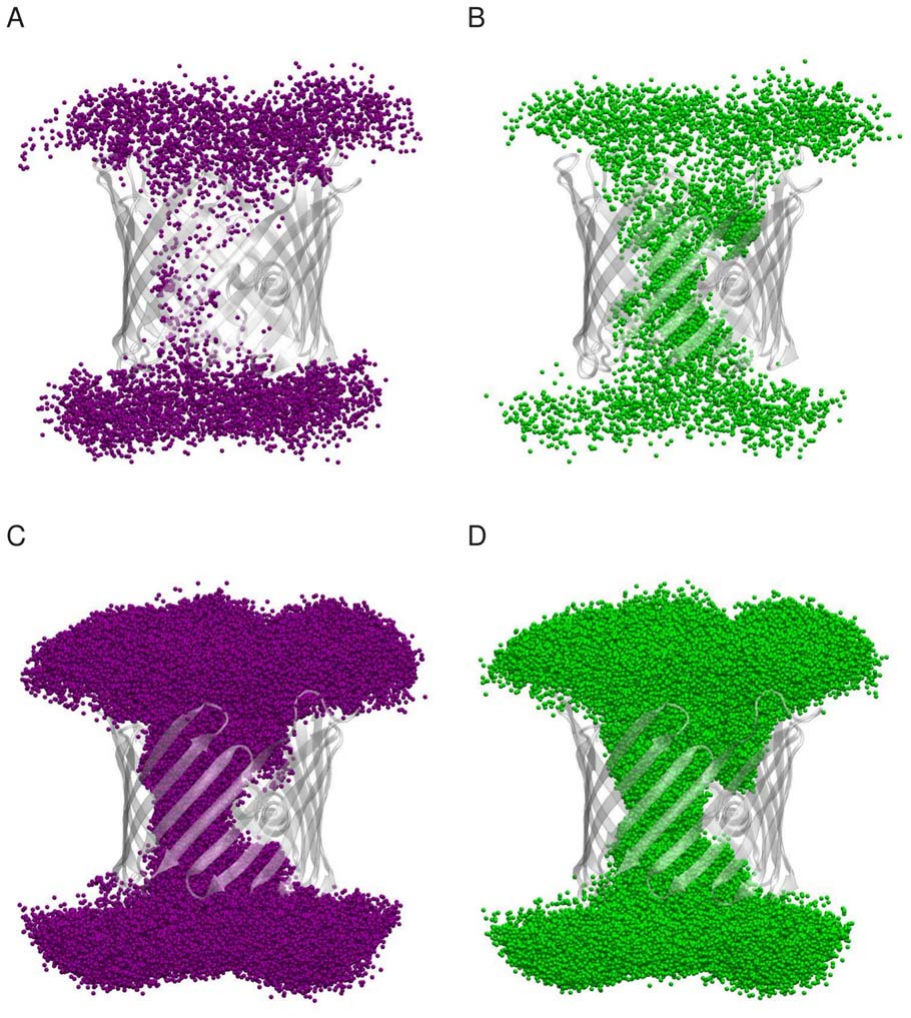
Ion pathways followed by K^+^ and Cl^−^ through mVDAC1 channel. Positions of the K^+^ (purple; A, C) and Cl^−^ (green; B, D) ions within 10 Å of the protein residues are superimposed using 2000 snapshots extracted every 500 ps from the 100 ns trajectories at 0.1 M (A, B) and 1.0 M (C, D). The protein is shown as a transparent-white cartoon.

The superimposition of the ion configurations extracted from the trajectories at 0.1 M and 1.0 M shows that, in contrast to OmpF [43], [44], [46], no specific pathways are followed by potassium or chloride as they cross the pore (Figure 4).

#### Ion pairing facilitates the transport of potassium through the pore

The frequency of potassium-chloride ion pairing was calculated as a function of the position along the pore axis for the 0.1 M and 1.0 M KCl trajectories (Figure S5). Due to a larger number of ions, ion pairing becomes more frequent at high concentration throughout the whole system and in particular across the channel rationalizing the observation that K^+^ translocation is more frequent at 1.0 M KCl than at 0.1 M KCl. In OmpF enhanced ion pairing was observed only in the constriction zone [44]. The difference in ion pairing between OmpF and VDAC may be interpreted by the existence of well separated diffusion pathways for potassium and chloride in OmpF and their absence in mVDAC1.

At high KCl concentration a marked decrease in ion pairing is observed at the membrane-protein interface relative to the bulk, likely to be due to interactions detected between potassium and negatively charged residues of the protein loops (Figure S5). Inside the pore ion pairing occurs with a higher frequency relative to the membrane-solvent interface and the bulk suggesting that potassium needs chloride to travel through the pore. This effect is more pronounced in the 0.1 M KCl simulation.

#### The charge distribution inside the mVDAC1 pore causes the attraction of anions

**Figure 5:**
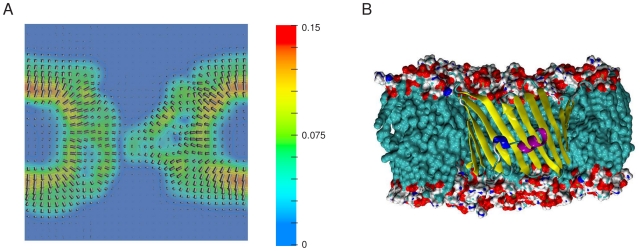
The electric field in mVDAC1 pore. (A) Cross section along the z axis through the channel. The orientation of the time-averaged electric field computed using the first 0.1 M KCl trajectory is depicted by arrows. Its magnitude is given as a color scale shown on the right hand side. The electric field was calculated using the PMEPot [83] module of vmd [76] and is shown using OpenDX (http:/www.opendx.org). The field shows that at the two entrances of the pore chloride ions are attracted and cations are repelled. (B) Representation of the orientation of mVDAC1 as in the depicted cross section. A molecular-surface rendering and a ribbon representation are used for regions of the lipids and of the protein, respectively, located behind the plane of the cross section.

We calculated the time-averaged electric field map using our MD trajectories (Figure 5). The electric field created by the protein indicates the existence of an electrostatic force attracting anions from both sides of the pore. As in OmpF [45] a transversal field arises in the constriction zone of the channel.

We also computed the average water dipole orientation along the pore (z) axis (Figure S6). In the bulk, no preferential orientation of the water dipoles is observed. The orientation of the water molecules is perturbed near the lipid bilayer and the protein. In the membrane headgroup region, the water molecules are orientated perpendicular to the membrane surface with their dipole positive charge pointing towards the negatively charged phosphate atoms of the lipid molecules. Within the channel, the water dipole orientation varies depending on its location along the pore axis. Entering the pore from the lower leaflet (z<0), a positive charge density is experienced by the water molecules, leading to a reorientation of the dipole. Just below the constriction zone, several negative charges spin round the orientation of the water dipoles. In the constriction zone, (Figure S6) the water dipoles are preferentially orientated perpendicular to the channel axis due to the occurrence of a transversal field (Figure 5). However, this effect is less pronounced compared to OmpF. At the upper opening of the pore, another density of positive charges induces a reorientation of the dipoles. The variations inside the channel are less pronounced at high KCl concentrations due to a screening of the fixed charges. However, at both salt concentrations, the orientation of the water dipoles is consistent with the computed electric field.

### Energetics of ion translocation

To determine whether VDAC electrostatic pattern favors the transport of anions we calculated the work needed to translocate an ion through the channel. This was done using two approaches: a continuum electrostatic calculation resorting to the solution of the Poisson-Boltzmann (PB) equation and a calculation of the energetics extracted directly from the MD simulations. In contrast to the MD approach, the PB method does not account for the dynamics of the protein nor for the ion-ion coupling and treat the solvent as a structureless continuum. The PB approach has however been shown to be valid for large pores such as VDAC [29], [49]–[51]. For a fairer comparison between the MD and the PB profile and as the permeation of chloride and of potassium do not follow defined paths (Figure 2, Figure 3), a 3D grid was used to calculate the PB profile (Figure S7 and Materials and Methods section).

#### Effect of bulk salt concentration

**Figure 6:**
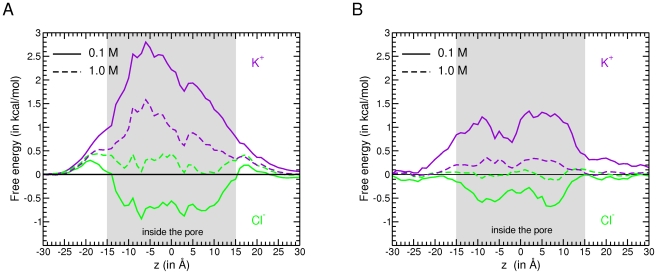
The ion translocation profile through the pore is concentration dependent. (A) The electrostatic free energy profile computed using the PB approach (B) The free energy profile computed using the MD simulations. PB and MD permeation profiles were determined along the axis of the pore for K^+^ (purple) and Cl^−^ (green), in a KCl concentration of 0.1 M (solid line) and 1.0 M (dashed line).

The overall profile of the MD and the PB free energies are very similar (Figure 6) albeit their magnitude differs in particular for K^+^ at 0.1 M. These discrepancies may arise from the differences in the PB and MD approaches. Ion pairing which has been shown to be important in the pore (Figure 5) is not accounted for in the PB calculations, since only a single ion is explicitly treated.

At 0.1 M the two profiles show that the transport of chloride is strongly favored relative to that of potassium. Two local minima are found for Cl^−^ which correspond to locations below and above the N-terminal helix. They are found to be more pronounced in the MD profile. This contrasts with the single minimum identified in a previously published PB profile [29]. The difference could be due to the reduction of the profile from a three- to a one-dimensional path calculation. At 1.0 M, the energy wells for chloride are less pronounced in the PB and MD profiles. For potassium, the energy barrier is significantly reduced at 1.0 M relative to 0.1 M, promoting the translocation of K^+^ at high concentrations.

#### Important specific residues

We identified nine charged residues that are close to low free energy values in the PB and MD profiles at 0.1 and 1.0 M concentration (Table 2). All nine residues are positively charged: Lys12, Lys20, Lys32, Lys96, Lys113, Arg139, Lys200, Lys236, and Lys256.

**Table 2:** Conservation analysis of residues important for the electrostatic signature of the protein.

Residue	Conservation (%)	Exchanged to (%)							
		Negative (D/E)		Positive (R/K)		Polar (T/W/S/N/Q/Y/C/H)		Other (A/G/I/L/M/P/V/F)	
**Lys12**	93.3			R	6.7				
**Lys20**	81.5			R	17.0	Q	1.5		
**Lys32**	59.3			R	3.7	T/S/Q/N/Y/H	37.0		
**Lys96**	95.6	E	0.7	R	0.7	T/Q/H	3.0		
**Lys113**	91.9			R	2.2	H	0.7	V/A/I	5.2
**Arg139**	7.4	E/D	8.9			N/H/Y/T/S	77.0	V/L	6.7
**Lys200**	8.9	D/E	31.9	R	4.4	S/N/H/T/Y/C	13.3	P/A/L/G	40.0
**Lys236**	72.6			R	26.7			P	0.7
**Lys256**	36.3			R	1.5	T/S/H	29.6	L/F/V/I/M	32.6

These residues were identified as being close to low energy regions in all MD and PB profiles of Cl^−^. Only charged residues were considered, since they have the highest influence on the electrostatics. For each identified residue the conservation and exchange rate based on a MSA of 135 VDAC sequences are listed. For Lys200 the complete percentage does not lead to 100% since there were two gaps found at this position in the MSA. The residue numbering refers to mVDAC1.

To investigate the potential conservation of these residues we built a multiple sequence alignment (MSA) using 135 sequences. Interestingly most of these VDAC sequences selected from plants, animals and fungi carry an overall positive charge (Table S1), with an average value of 3.0 and a standard deviation of 2.0 which is consistent with the idea that VDACs from different phylogenetic lineages are all anion-selective [7], [8], [52]–[55].

The conservation analysis revealed that five of the nine positively charged residues identified in the energy profiles (Lys12, Lys20, Lys96, Lys113, and Lys236) are conserved or at least functionally conserved (Table 2). Residues that are conserved in a protein family are expected to be critical for function, structure or stability. Mutagenesis experiments reported that the introduction of a negatively charged residue at position 20 and 96, respectively (numbering refers to mVDAC1) affects the selectivity of *S. cerevisiae* VDAC [52]: the K20E VDAC mutant was found to be slightly cation selective and the K96E variant is less anion selective than the *wild-type*. To our knowledge the other three residues have not been investigated so far.

### The selectivity of plant VDAC32 depends on the ionic strength

**Figure 7:**
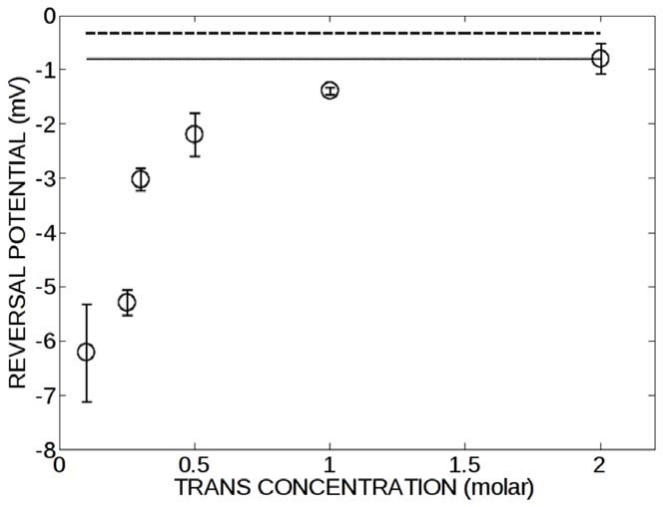
Effect of ionic strength on the bean VDAC32 reversal potential. The reversal potential (in open circles) is plotted against the salt concentration of the trans compartment. The concentration ratio was kept constant at a value of 2.0. The reversal potential calculated using the voltage GHK equation (with the P_Cl_/P_K_ = 1.10 at a concentration ratio 2/1 M KCl (trans/cis)) is shown as a solid line. The reversal potential calculated using the Planck equation (diffusion coefficient of ion in solution D_Cl_/D_K_ = 1.04) is shown as a dashed line.

Previous studies in our group showed that bean VDAC32 features electrophysiological properties, i. e. anion selectivity, single channel conductance and voltage-dependent gating, similar to those of mammalian VDAC1 [9], [37]. Several reports have also pointed out that plant and mammalian VDACs are likely to adopt a similar structure [24], [55], [56]. All these data prompted us to investigate the salt dependence of bean VDAC32 selectivity to assess the conservation across species of our proposed ion permeation mechanism. We thus performed reversal potential measurements on bean VDAC32. The reversal potential (zero current potential) measured in the presence of a salt concentration difference on both sides of the membrane is a widely used measure to estimate the selectivity of ion channels. In all experiments the concentration ratio between the trans and the cis compartment was maintained constant, the trans concentration being twice that of the cis compartment. Under these conditions a negative reversal potential indicates a preferred selectivity for chloride over potassium. Our results indicate that the VDAC32 selectivity towards chloride increases as the ionic strength of KCl decreases (Figure 7).

We also calculated the reversal potential using two well-known models: the Goldman-Hodgkin-Katz (GHK) model and the constrained liquid junction model. The GHK model assumes a constant electric field across the pore and the constrained liquid junction model implies a condition of electroneutrality (C_K_ = C_Cl_ = C_KCl_). For a symmetric monovalent salt the GHK potential is given by:
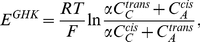
(1)where R is the gas constant, T the absolute temperature, F the faraday constant, α is the permeability ratio (P_C_/P_A_), 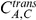 and 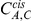 are the concentration of ion species (subscript A and C refer to anions and cations, respectively) in the trans and cis compartments, respectively. The constrained liquid junction potential (E^planck^) is given by the Planck equation:
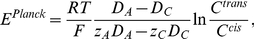
(2)where *z* is the ionic charge, *D* is the diffusion coefficient, C^trans^ and C^cis^ are the concentrations of the ionic species and subscript *A* and *C* refer to anions and cations, respectively. Both equation [1] and [2] are valid for a neutral channel. Figure 7 shows that the reversal potential calculated with either equation [1] or [2] does not vary with the ionic strength when the concentration ratio is constant. At high ionic strength the measured reversal potential for VDAC32 is close to that predicted by E^Planck^ suggesting that most of the fixed charged are screened.

## Discussion

### mVDAC1 structure reveals a concentration dependent preference for anions

The overall structure of mVDAC1 remains stable in all MD simulations. The dynamic behavior of the protein conformations compares rather well with the experimental data which sustains the adequate conformational sampling of the trajectories.

Our simulations were performed under equilibrium conditions, in symmetric ion concentration and with no transmembrane potential. Although in these conditions no net flux occurs several ion translocation events were monitored along our trajectories. This may raise a problem when it comes to compare to experimental data which are obtained in non equilibrium conditions. VDAC permeation properties were however also characterized upon small values of voltage or concentration gradient and are thus close to our simulation conditions (Figure 7 and [14]).

Our simulations clearly show that anions are more frequently found inside mVDAC1 and cross the pore more frequently than cations (Table 1) in agreement with experimental observations and a previous theoretical study. The MD trajectories furthermore reveal that the N_Cl−_/N_K+_ ratio inside the channel depends on the bulk salt concentration (Figure 3). This ratio is significantly higher at low relative to high salt concentrations.

The effect of the protein electrostatics on the ion permeation is important at low concentration and diminishes with increasing concentration due to a screening of the protein charges. According to our MD trajectories, the electrostatic effect is totally screened at a KCl concentration higher than 0.8 M (Figure 3) leading to a constant N_Cl−_/N_K+_ ratio inside the protein of about 1.5. The screening of the electric field created by the charges lining the VDAC pore at a high salt concentration is likely to be facilitated by the large pore size of VDAC.

The electric field computed using our trajectories clearly show that both entrances of the pore attract anions and repel cations. In the constriction zone, at about the middle of the pore, a transversal electric field occurs (Figure 5) that causes a local preferential orientation of the water molecule dipoles (Figure S6). Other factors, which are important for the selectivity in OmpF [43], [44], such as distinct translocation pathways, specific long-lived protein-ion interactions, and differences in translocation times between Cl^−^ and K^+^ do not appear to play a role in mVDAC1 ion translocation process. Further evidence for the protein electrostatics as the main determinant of the pore selectivity is shown by the similarity of the PB and MD free energy surface profiles for the chloride and potassium translocation through VDAC. At 0.1 M a large energy barrier prevents potassium from crossing the pore and two energy wells are observed for chloride whereas at 1.0 M the energy barrier for potassium is significantly reduced facilitating the translocation of K^+^ and the energy wells for chloride are less deep.

Moreover, we identified several key residues close to low free energy regions, which are likely to be important for mVDAC1 electrostatic signature. These residues are all positively charged and several of them are conserved across different eukaryotic species from plant to yeast and human (Table 2). Two of these residues (Lys20 and Lys96) investigated by mutagenesis have been reported to be important for VDAC selectivity [52]. We propose that the molecular mechanism determining VDAC selectivity is prevalent in the VDAC family.

### Importance of the N-terminal region for the translocation process

MD and BD simulations have been performed recently to study the electrophysiological properties of the NMR structures of human VDAC1 at different voltage values and in the absence or in the presence of one concentration gradient value [30], [31]. These studies also highlight the importance of the protein electrostatics for VDAC selectivity. The N_Cl−_/N_K+_ ratio inside the hVDAC1 pore obtained at 1.0 M KCl in the absence of a transmembrane potential is similar to our value on mVDAC1 [30], [31]. The translocation energy profiles of Cl^−^ and K^+^ however differ. In hVDAC1 there is a significant free energy barrier at the two entrances of the pore of about 1.5 and 2.0 kcal/mol for Cl^−^ and K^+^, respectively [30], and a free energy well, small for Cl^−^ and deep for K^+^ is also found at the level of the constriction zone [30]. In contrast, in our simulations, no energy barrier for chloride occurs in mVDAC1 suggesting that the permeation occurs spontaneously. Another difference is that in hVDAC1, even so specific pathways are also not detected as in mVDAC1, strong interactions between K^+^ and a few protein residues, namely Asp16, Asp30, Glu84 and Asn207, were identified. These interactions were proposed to hinder crossing events of potassium relative to chloride. Such interactions are not observed in our MD trajectories of mVDAC1 (Figure S4).

All these disparities may arise from structural differences between the N-terminal region of the Xray mVDAC1 and NMR hVDAC1 structures (Figure 1). The mVDAC1 structure shows a wide open pore, which is restricted by the N-terminal helix that leans onto the barrel wall. In contrast, the pore in the hVDAC1 structure is narrower as the N-terminal segment occupies about the middle of the pore. These structural differences may also change the solvent accessibility surface of a series of residues lining VDAC pore.

The comparison between the translocation energetics of Cl^−^ and K^+^ through the mVDAC1 and hVDAC1 structure stresses the importance of the pore local structure for its selectivity. In particular the position of the N-terminal region within the channel seems to influence the functional properties of the pore. This observation concurs with experimental data that reported the importance of the N-terminal region in VDAC voltage-gating [25], [28], [57], [58].

### Plant VDAC selectivity buttresses the proposed molecular mechanism

The importance of electrostatic interactions for the ion selectivity has already been demonstrated previously for the bacterial OmpF [40], [41], [43], the fungi VDAC [14], [39] and nanopores [59]. In contrast, it has been reported that *Dictyostellium* VDAC selectivity behaves like a neutral pore [13]. The discrepancy between the observations made on *N. crassa* and *Dictyostellium* could be a consequence of the simultaneous change of both concentration ratio and ionic strength in the experiment carried out on *Dictyostellium*.

We investigated the electrophysiological properties of bean VDAC32 that features functional and structural properties similar to mammalian VDAC1 [9], [24], [37], [55]. Experiments performed using a constant concentration ratio showed that the selectivity of this channel varies nonlinearly with the ionic strength. VDAC32 reversal potential becomes more negative as the ionic strength decreases indicating an increase of selectivity toward anions owing to the net positive charges of the pore. This result indicates that the fixed charges of the pore in VDAC32 play a significant contribution to the channel ion selectivity and are consistent with the observations extracted from our simulation data on mVDAC1. Both the GHK potential (Equation [1]) and the Planck equation (Equation [2]) failed to model the obtained salt dependence of the measured VDAC selectivity. These approaches neglect electrostatic effects that might occur in large nanometric pores. They describe the electrical potential difference that would set up across a neutral channel in the presence of a salt gradient, due to the fact that ions have a different diffusion coefficient. The GHK and the Planck equation are thus valid approximations at high ionic strength, viz. when the charges of the channel are screened by counter ions and that the channel is electrically neutral. At low ionic strength, however, a significant deviation of these two models occurs. Our theoretical results on mammalian VDAC1 are consistent with the experimental data obtained on plants (Figure 7) and fungi [10], [35]. Altogether these results suggest that the mechanism for ion permeation proposed in this study may be conserved in the VDAC family.

## Materials and Methods

### Simulation setups and protocols

The initial configuration was built starting from the mVDAC1 crystal structure (pdb code 3emn) [19]. pKa calculations were performed using the program propKa 2.0 [60]. Accordingly, all ionizable residues including Glu73 were taken in their standard protonation states corresponding to pH 7. Glu73 belongs to the β-barrel and faces the membrane. There is however evidence that this residue should be in its ionized state [19], [61].

The protein was inserted in a lipid bilayer using the CHARMM-GUI web server (http:/www.charmm-gui.org) [62]. The channel was centered at z = 0 and oriented guided by the OPM (Orientation of Protein in Membranes) database tool [47]. The N- and C-termini were positioned in the lower leaflet with z<0. 1-palmitoyl-2-oleoyl-*sn*-glycero-3-phosphoethanolamine was used to model the lipid environment. KCl was added at a concentration of 0.1 M. The net excess charge of VDAC was counterbalanced by addition of three chloride ions. In total the molecular system comprises about 42000 atoms including 180 lipid and ∼9000 water molecules. Its size is about 80×85×76 Å^3^. The all-atom CHARMM27 force field [63], [64] with CMAP corrections [65] was used to describe protein, water, and ion atoms and a united atom force field [66] described the lipid molecules.

The system was carefully equilibrated in three steps: first, a 50 ns equilibration of the fixed protein was performed to remove possible clashes between the protein and its environment without altering the protein structure. Second a 20 ns equilibration with the protein backbone constrained was carried out to remove possible bad contacts between protein side chain atoms, and last unrestrained equilibration was performed for 20 ns.

All MD simulations were carried out in the isothermal-isobaric ensembles at 300 K with the program NAMD [67] in the absence of a transmembrane potential. Long-range electrostatic interactions were described using the particle-mesh Ewald method [68]. A smoothing function was applied to truncate short-range electrostatic interactions. The use of the Verlet-I/r-RESPA multiple time-step propagator [69] allowed to integrate the equation of motions using a time step of 2 and 4 fs for short- and long-range forces, respectively. All bonds with hydrogens were constrained using the Rattle algorithm [70].

### MD simulations of mVDAC1 in different salt concentrations

After equilibration of the system in 0.1 M KCl, two 50 ns trajectories were produced following two different strategies: In one of the simulations, the ionic distribution was that of the equilibrated system. In the other simulation, a new distribution of the ions was generated and the system was equilibrated again for 5 ns.

After 50 ns of production run, the ionic salt concentration was adjusted to 0.2 M KCl using these two different set-ups. A 5 ns equilibration and a production run of 15 ns were performed. The same protocol was used to increase the KCl concentration by steps of 0.2 M until a KCl concentration of 1.4 M was reached.

At 1.0 M KCl two 50 ns trajectories were produced. One of the simulations was performed starting with a completely new random distribution of ions. A longer equilibration time of 15 ns was carried out for this system.

Whenever needed for the analysis the two independent 50 ns trajectories at 0.1 or at 1.0 M KCl concentration were combined. Such a combination is reasonable, since the two individual simulations behave similarly with respect to dynamical and functional properties (Table 1 and Figure S1).

### Free energy profile of ion permeation through mVDAC1

#### Electrostatic free energy profiles based on the crystal structure

Ion transfer free energies were calculated at 300 K for a chloride-sized anion and a potassium-sized cation through the mVDAC1 crystal structure pore (pdb code 3emn; [19]) at 0.1 M and 1.0 M KCl. A grid with a spacing of 1 Å was used with a size of 40×40×70 Å^3^ (Figure S7). Grid point clashes with the protein and/or the membrane atoms were subsequently removed leading to a total of about 72 000 grid points. At each point the electrostatic free energy corresponding to the transfer of an ion from solution into the protein/lipid environment was calculated using a PB approach producing a 3D profile. Averaging the energy values at constant z value resulted in a one dimensional energy profile.

In the PB approach, ions, water and lipids are implicitly described (Figure S7). The thickness of the hydrophobic part of the membrane was set to 25 Å following the OPM recommendation [71]. A dielectric constant of 2 and 4 was used to depict the hydrophobic part of the membrane and the protein, respectively. The water and the membrane head group region were represented by a dielectric of 80. All electrostatic calculations were done with the program APBS (version 1.2.1) [72]. The PB equation was solved using a finite difference method on a 300 Å×300 Å×300 Å grid with two levels of focusing. At the last step the grid spacing was 0.8 Å. Partial charges were taken from the CHARMM force field [63], [64].

#### Free energy profile using the MD trajectories

The averaged multi-ion free energy profile along the pore axis was calculated at 300 K in presence of 0.1 M and 1.0 M KCl combining for each concentration the two 50 ns MD simulations. The free energy of an ion species *i* inside the channel at a position *z* was calculated using the following equation [73]:
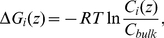
(3)where R, T, C_i_(z) and C_bulk_ are the gas constant, the temperature, the concentration inside the pore at z, and bulk concentration of the ionic species *i*, respectively. This method has been successfully used for the determination of the free energy profile of small compounds inside protein pores [74], [75] and also of ions inside hVDAC1 [31]. A 3D energy profile was also calculated using the MD trajectories by solving eq. 3 on a 3D grid as in the PB approach.

Based on the 3D free energy profiles calculated with PB and MD at either 0.1 M or 1.0 M KCl residues close to low energy regions were identified. A residue was selected if the distance between the oxygen or nitrogen atom of the residue side chain and grid points having values within 1% of the lowest energy profile values was inferior to 4 Å. The one dimensional energy profiles were used to study the effect of the ionic concentration on the ion transfer.

### MD simulations of the 0.1 M and 1.0 M KCl solution

Two MD simulations were carried out on a 0.1 M and 1.0 M KCl solution using NAMD. The system consisted of about 48.000 atoms including 62 and 612 ions in the 0.1 and 1 M KCl solution respectively in a cubic box with a side length of 80 Å (initial size). After equilibration of 2 ns, a data collection run of 10 ns was produced and used to compute the radial distribution function (RDF) of water around potassium and chloride (Table S2) using vmd [76], [77].

The position of the first peak in the RDF is 3.54 and 3.76 Å at 1.0 M for K^+^ and Cl^−^, respectively. Our hydration shell radii are in good agreement with earlier MD simulations [44] even so the authors of this study used a slightly different water model. The coordination number after the first RDF peak equals to 6.3 and 7.3 at 1.0 M (Table S2), values in good agreement with the experimental estimate of 6 to 8 water molecules [78]. Due to the smaller number of ions in the 0.1 M system the RDF position of the peaks and the coordination number are marred with large fluctuations (Figure S2). They however show the same trends as at 1.0 M (Figure S2). Thus only the results obtained at 1.0 M were used for analysis.

Ion pairing can be classified into contact ion pairs (CIP) and solvent separated ion pairs (SSIP) [79]. The parameters needed to describe the CIP and the SSIP are the positions of the first and second minima in the RDFs of the water molecules around potassium and chloride ions. In our simulations the CIP spans a cation-anion distance between 0 and 3.95 Å and the SSIP ranges between 3.95 and 6.37 Å. These values are in good agreement with an earlier MD study, in which the CIP stretches out to 4.00 Å and the SSIP to 6.45 Å [44]. We used the maximal value of the SSIP to count the number of ion pairs in our trajectories.

### Conservation study

Based on a set of 135 sequences including 60 animal, 31 fungi and 44 plant VDACs a multiple sequence alignment (MSA) was constructed using the program clustalX [80]. The sequences were found using the BLAST [81] algorithm as implemented on the NCBI webpage [82] starting from the sequences of the mammalian (mVDAC1), fungus (*S. cerevisiae* VDAC), and plant (*P. coccineus* VDAC32) VDAC. All sequences are listed in Table S1. The MSA was used to investigate the conservation of functionally important residues identified in our MD/PB calculations on mVDAC1. A residue was considered as conserved if this residue or a residue with similar physicochemical properties is found in over 90% of the sequences at the same position. The theoretical charge of each VDAC was calculated based on its sequence assuming all titratable residues in their standard protonation state at pH 7.

### Electrophysiological experiments with plant VDAC32

VDAC32 from *P. coccineus* was purified as described previously [9]. The experimental procedure used for electrophysiology was described recently in detail [37]. Briefly, VDAC was reconstituted in planar lipid bilayers formed from soy lipid extract (Avanti) dissolved in hexane to a final concentration of 2% (w/v). Planar lipid bilayers resulted from the folding of two lipid monolayers over a hole (135 µm in diameter) made in a 25 µm thick Teflon partition that separated two Teflon experimental chambers. Before each experiment the partition was treated with a solution of hexane/hexadecane (2.5%, v/v) to increase its oleophylicity. Ag/AgCl electrodes connected in series with a salt bridge (1 M KCl in 1% agar) were used to connect the experimental chambers to the electronic equipment. The trans compartment is defined as the one connected to the ground and the voltage was applied to the cis compartment. For channel reconstitution into a planar lipid bilayer, proteins were added to the cis compartment. Data were measured using a BLM 120 amplifier (BioLogic, France), filtered at 300 Hz (5-poles linearized Tchebichev filter), digitized at 44.4 kHz with a DRA 200 interface (BioLogic, France) and stored on CD for further processing using a homemade program written in the MATLAB environment (The MathWorks, Natick, MA).

To assess the role of the ionic strength on VDAC32 selectivity, its reversal potential was first set to zero in presence of identical KCl concentration on both sides of the membrane. Then, the cis compartment was perfused three times its volume with a KCl solution twice less concentrated and the change of reversal potential (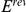) was recorded.

## Supporting Information

Figure S1 **Average fluctuations of mVDAC1 during the MD simulations.** The backbone RMSF of the first (solid line) and the second (dashed line) 50 ns MD simulation in 0.1 M (brown) and in 1.0 M KCl (black) are shown as well as the crystallographic B factors (yellow). The β-strands are highlighted in grey stripes. The fluctuations of mVDAC1 are similar in all four simulations.
(TIFF)

Figure S2 **Ion diffusion through the pore.** The dynamics of the chloride ions (A) and potassium ions (B) along the pore (z) axis is depicted as a function of the simulation time. Each ion is represented by a different color. All data were extracted from the first 0.1 M MD trajectory.
(TIFF)

Figure S3 **Dehydration of Cl^−^ and K^+^ ions during pore crossing.** The time-averaged number of water and protein residues solvating chloride (A) and potassium (B), respectively, are depicted across the pore axis (*z*) in 0.1 M (brown) and 1.0 M KCl (black). Chloride and potassium ions are only slightly dehydrated throughout the permeation process.
(TIFF)

Figure S4 **Interactions between ions and protein residues.** No long-lived interactions between Cl^−^ (A, B) or K^+^ (C, D) and protein residues are observed at 0.1 M (A, C) and 1.0 M (B, D) in the 100 ns MD trajectories. An interaction is marked by a tick. The first and the second 50 ns correspond to different simulations and are highlighted by different background colors (first simulation in white, second simulation in gray).
(TIFF)

Figure S5 **Ion pairing inside the pore.** Ion pairing including the CIP and SSIP increases inside the pore as shown by the higher number of potassium ions in interaction with chloride ions in the 0.1 M (brown line) and 1.0 M (black line) MD trajectories.
(TIFF)

Figure S6 **Water dipole orientation extracted from the MD simulations.** (A) The orientation of the averaged water dipole shown for 0.1 M KCl (in brown) and 1.0 M KCl (in black) depends on the position along the pore (z) axis. (B) For different z values, the probability distribution of the dipole orientation is given.
(TIFF)

Figure S7 **Electrostatic energy calculations.** (A) In the PB approach the protein (in yellow) is represented by fixed point charges and a low dielectric constant. The water and the hydrophilic part of the membrane (in pink) are represented by a high dielectric constant value. The hydrophobic part of the membrane (in grey) is represented by a low dielectric constant. (B) At each point of the grid with a spacing of 1 Å the electrostatic energy corresponding to the transfer of the ion from the solution to this point is calculated. Each point of the grid is shown as a small grey sphere and the protein is shown in cartoon.
(TIFF)

Figure S8 **Radial distribution function of chloride around potassium.** The radial distribution functions of chloride around potassium computed using the MD trajectories of 0.1 M (brown) and 1.0 M (black) KCl solutions are shown.
(TIFF)

Table S1 **VDAC sequences used for the MSA.** Sequences are listed following their species name, GenInfo identifier and isoform as given by the database (NCBI). In addition the theoretical charge at pH 7 was calculated for each sequence assuming that each titratable residue is in a standard protonation state at pH 7 thus using a charge of −1 for Asp and Glu, +1 for Arg and Lys, and 0 for all other amino acids.
(DOC)

Table S2 **Radial distribution properties computed from simulations of 0.1 M and 1.0 M KCl solutions.** The distances for the first and second peak (r_max_) and minima (r_min_), the height of the peak g(r_max_) and of the minima g(r_min_), as well as the integration number of the RDF up to its first (second) minimum (N(r_min_)) are listed. The RDF of potassium-chloride interaction at 0.1 M and 1.0 M show qualitatively the same behavior. However, at 0.1 M KCl the accurate determination of the potassium-chloride interaction parameters was prevented due to large fluctuations in the RDF (see Figure S8). All distances are given in Å.
(DOC)
