# Supplementary figures and images for: Concentration Dependent Ion Selectivity in VDAC: A Molecular Dynamics Simulation Study
Source: PLoS One. 2011 Dec 2;6(12):e27994. doi: 10.1371/journal.pone.0027994 (PMC3229507; doi:10.1371/journal.pone.0027994)

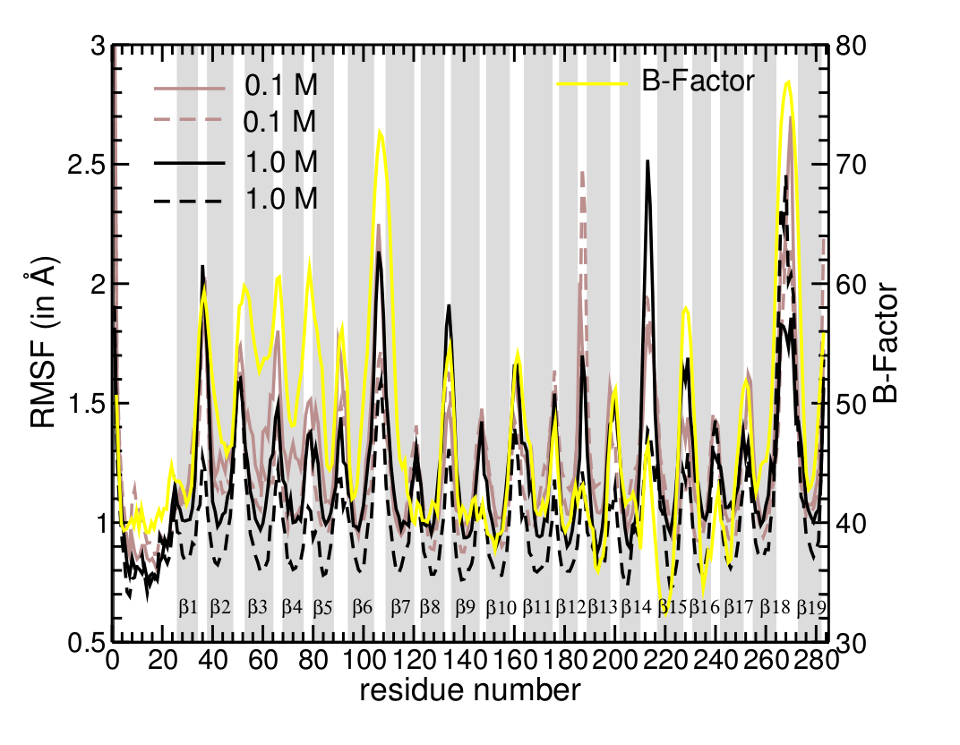

Supplement: Figure S1 — Average fluctuations of mVDAC1 during the MD simulations. The backbone RMSF of the first (solid line) and the second (dashed line) 50 ns MD simulation in 0.1 M (brown) and in 1.0 M KCl (black) are shown as well as the crystallographic B factors (yellow). The β-strands are highlighted in grey stripes. The fluctuations of mVDAC1 are similar in all four simulations. (TIFF) [file pone.0027994.s001.tiff]

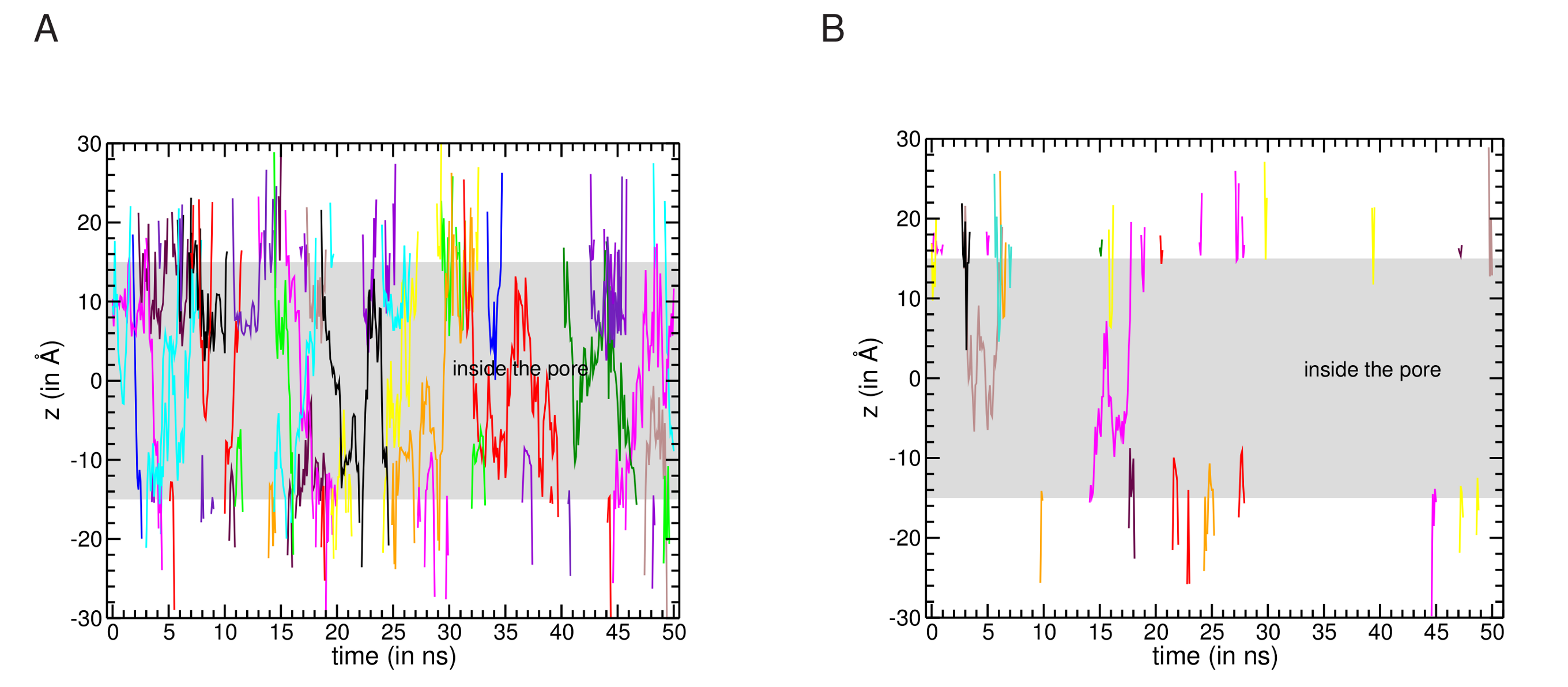

Supplement: Figure S2 — Ion diffusion through the pore. The dynamics of the chloride ions (A) and potassium ions (B) along the pore (z) axis is depicted as a function of the simulation time. Each ion is represented by a different color. All data were extracted from the first 0.1 M MD trajectory. (TIFF) [file pone.0027994.s002.tiff]

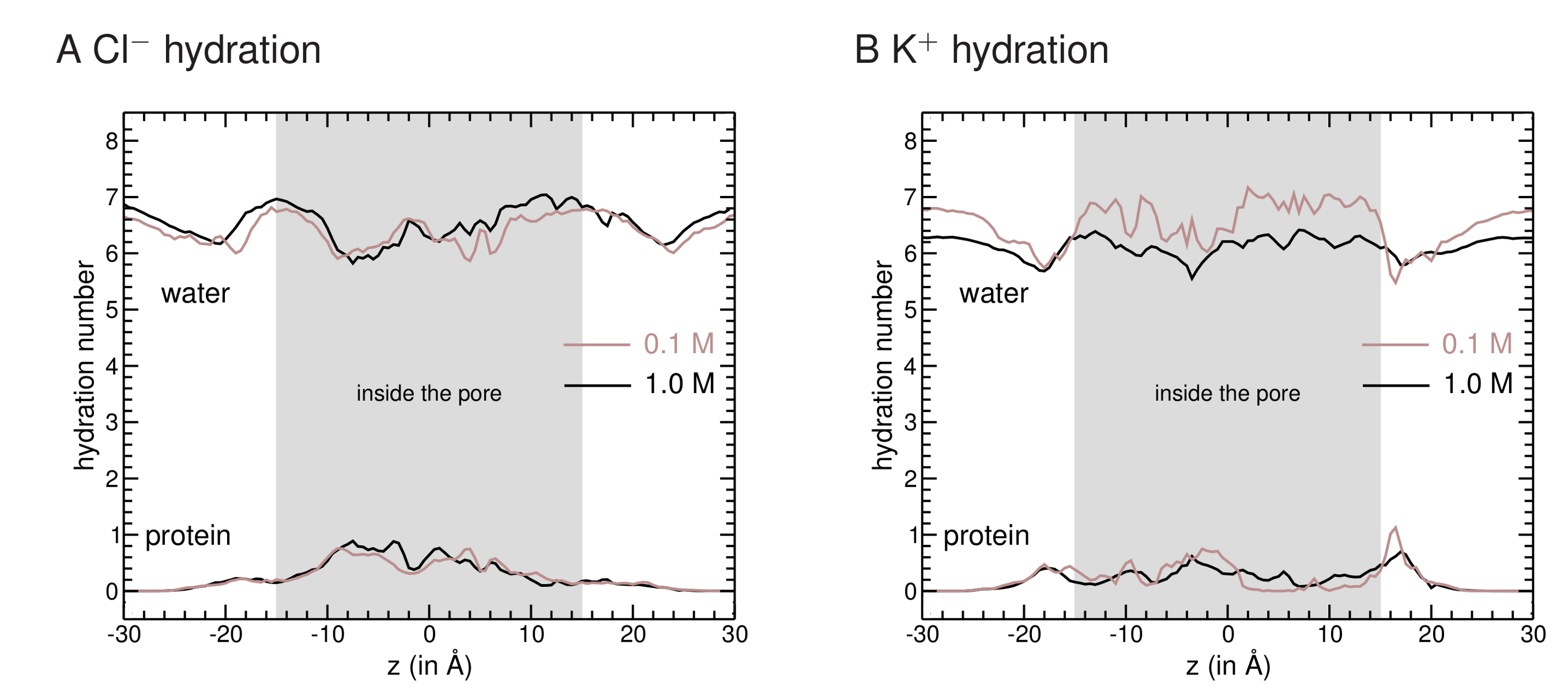

Supplement: Figure S3 — Dehydration of Cl− and K+ ions during pore crossing. The time-averaged number of water and protein residues solvating chloride (A) and potassium (B), respectively, are depicted across the pore axis (z) in 0.1 M (brown) and 1.0 M KCl (black). Chloride and potassium ions are only slightly dehydrated throughout the permeation process. (TIFF) [file pone.0027994.s003.tiff]

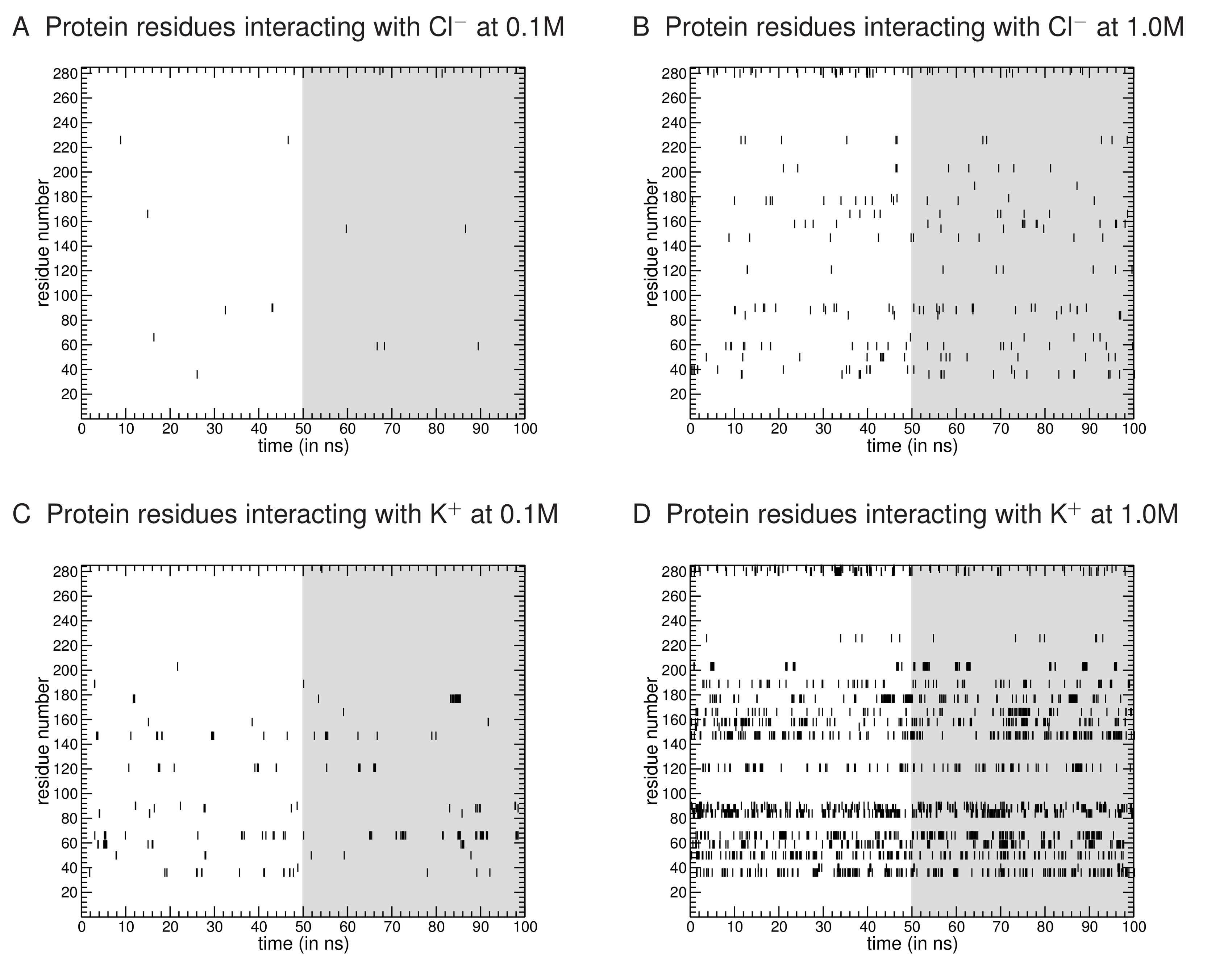

Supplement: Figure S4 — Interactions between ions and protein residues. No long-lived interactions between Cl− (A, B) or K+ (C, D) and protein residues are observed at 0.1 M (A, C) and 1.0 M (B, D) in the 100 ns MD trajectories. An interaction is marked by a tick. The first and the second 50 ns correspond to different simulations and are highlighted by different background colors (first simulation in white, second simulation in gray). (TIFF) [file pone.0027994.s004.tiff]

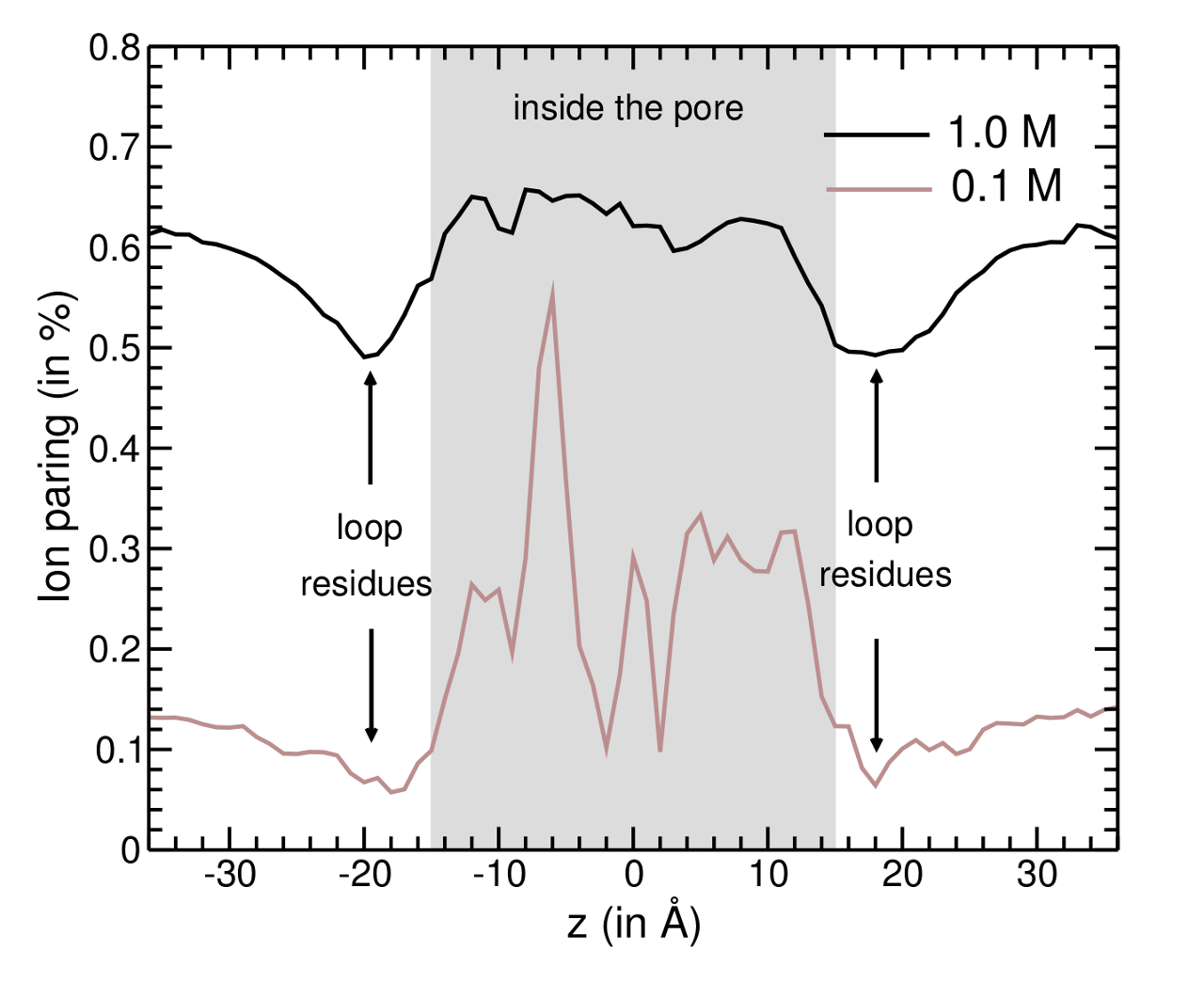

Supplement: Figure S5 — Ion pairing inside the pore. Ion pairing including the CIP and SSIP increases inside the pore as shown by the higher number of potassium ions in interaction with chloride ions in the 0.1 M (brown line) and 1.0 M (black line) MD trajectories. (TIFF) [file pone.0027994.s005.tiff]

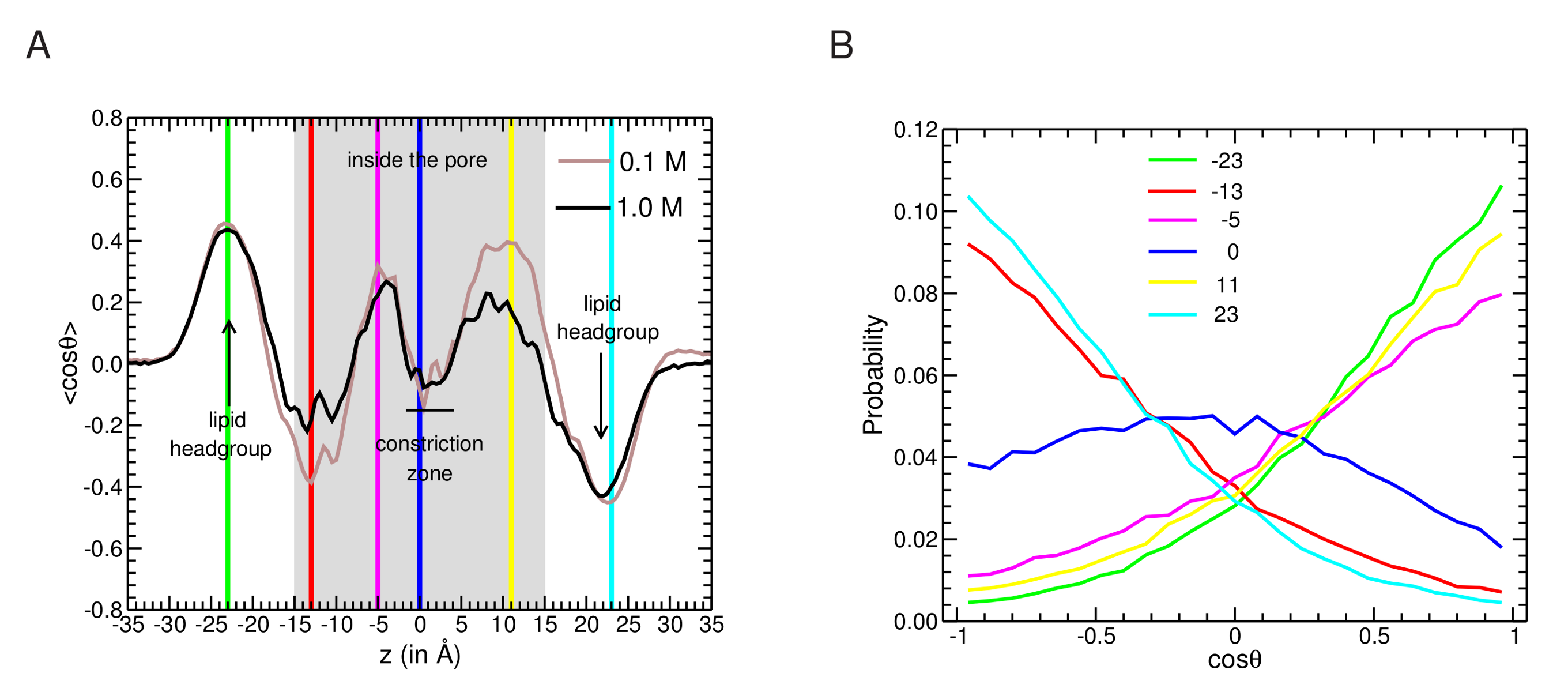

Supplement: Figure S6 — Water dipole orientation extracted from the MD simulations. (A) The orientation of the averaged water dipole shown for 0.1 M KCl (in brown) and 1.0 M KCl (in black) depends on the position along the pore (z) axis. (B) For different z values, the probability distribution of the dipole orientation is given. (TIFF) [file pone.0027994.s006.tiff]

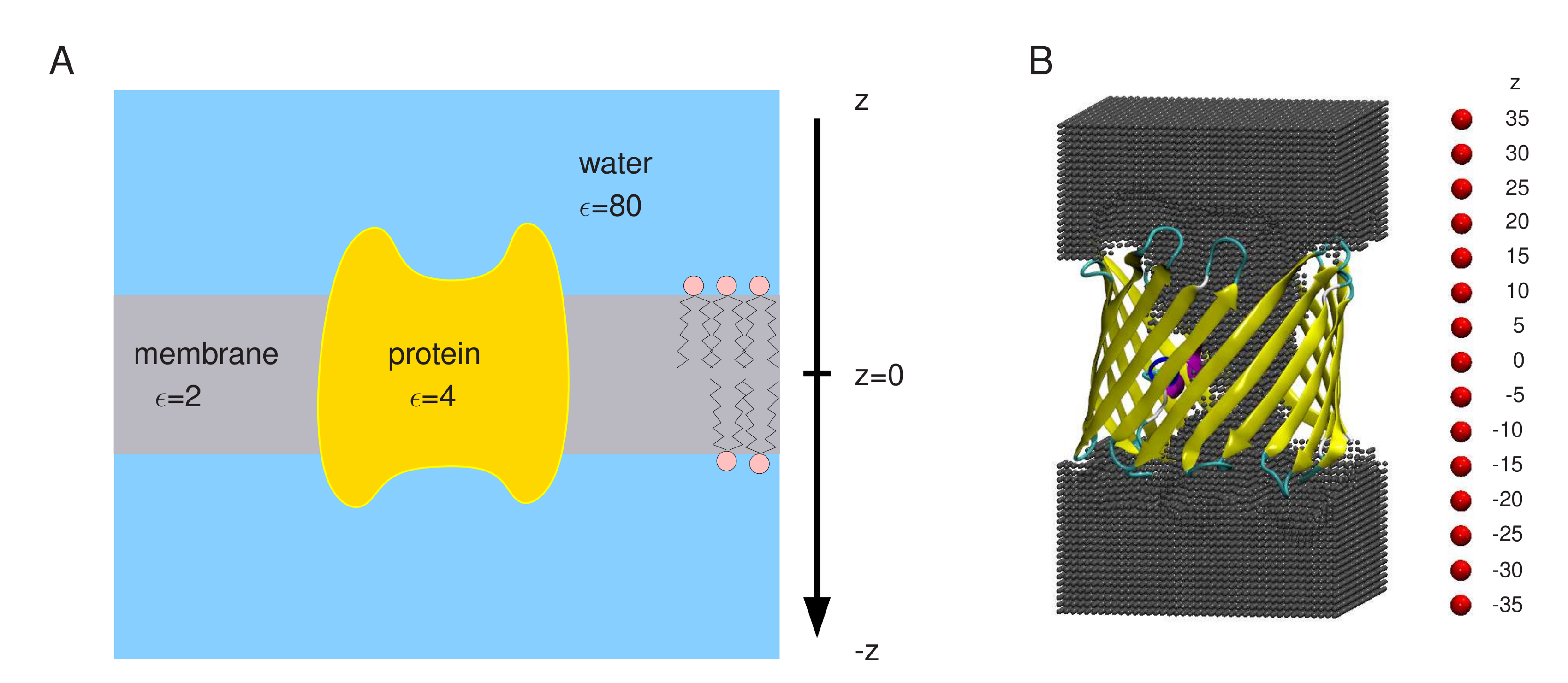

Supplement: Figure S7 — Electrostatic energy calculations. (A) In the PB approach the protein (in yellow) is represented by fixed point charges and a low dielectric constant. The water and the hydrophilic part of the membrane (in pink) are represented by a high dielectric constant value. The hydrophobic part of the membrane (in grey) is represented by a low dielectric constant. (B) At each point of the grid with a spacing of 1 Å the electrostatic energy corresponding to the transfer of the ion from the solution to this point is calculated. Each point of the grid is shown as a small grey sphere and the protein is shown in cartoon. (TIFF) [file pone.0027994.s007.tiff]

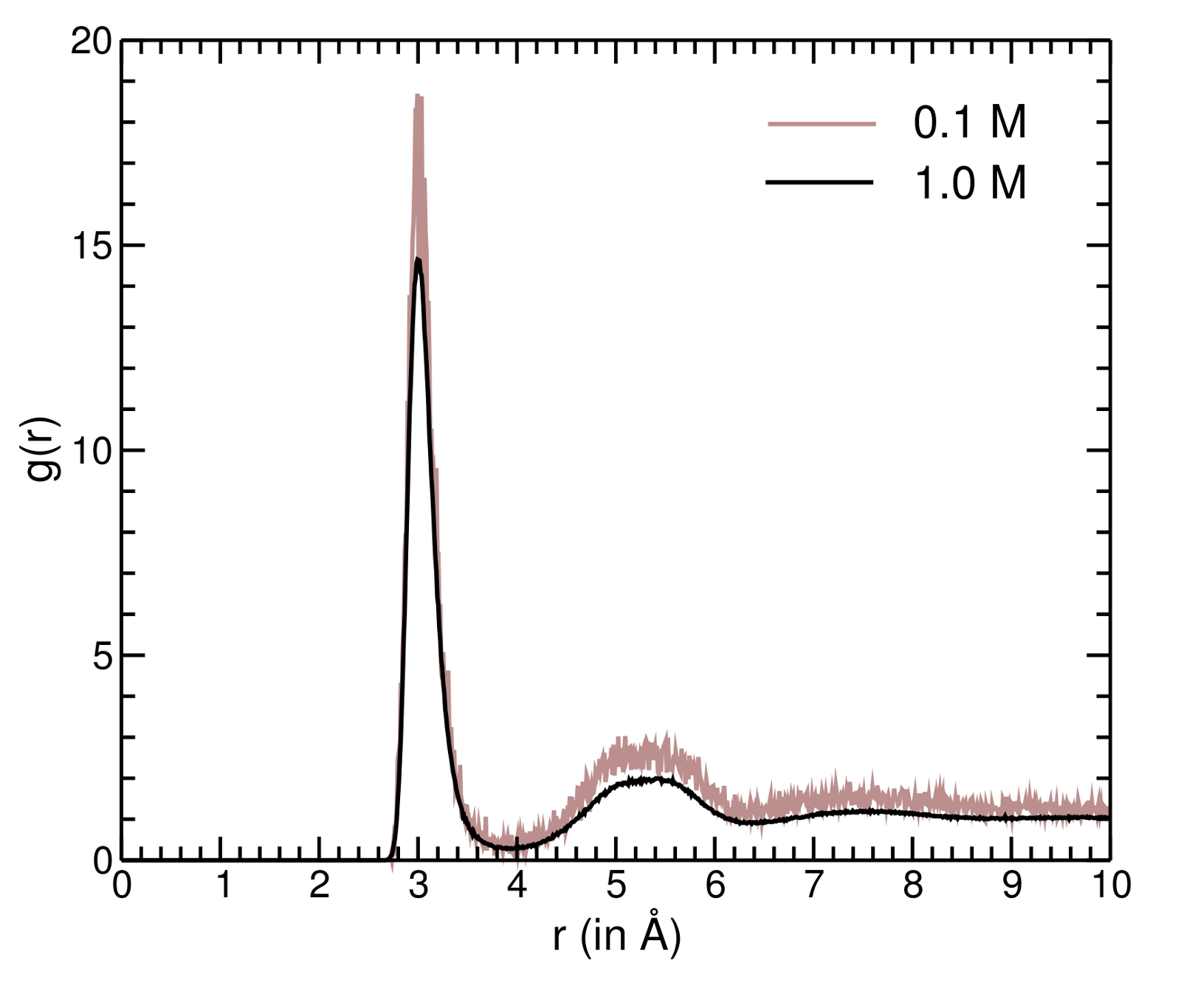

Supplement: Figure S8 — Radial distribution function of chloride around potassium. The radial distribution functions of chloride around potassium computed using the MD trajectories of 0.1 M (brown) and 1.0 M (black) KCl solutions are shown. (TIFF) [file pone.0027994.s008.tiff]
