# Supplementary material for: Concentration Dependent Ion Selectivity in VDAC: A Molecular Dynamics Simulation Study
Source: PLoS One. 2011 Dec 2;6(12):e27994. doi: 10.1371/journal.pone.0027994 (PMC3229507; doi:10.1371/journal.pone.0027994)
Supplement: Table S1 — VDAC sequences used for the MSA. Sequences are listed following their species name, GenInfo identifier and isoform as given by the database (NCBI). In addition the theoretical charge at pH 7 was calculated for each sequence assuming that each titratable residue is in a standard protonation state at pH 7 thus using a charge of −1 for Asp and Glu, +1 for Arg and Lys, and 0 for all other amino acids. (DOC) [file pone.0027994.s009.doc]

| Species | GenInfo Identifier | Isoform | Theoretical charge |
| --- | --- | --- | --- |
| *Aedes aegypti* | gi|157124666 |  | 3 |
| *Ajellomyces capsulatus G186AR* | gi|225560490 |  | 4 |
| *Ajellomyces dermatitidis SLH14081* | gi|261192617 |  | 4 |
| *Anopheles gambiae str. PEST* | gi|58391886 |  | 3 |
| *Arabidopsis thaliana* | gi|15232074 | VDAC1 | 3 |
|  | gi|15240765 | VDAC2 | 4 |
|  | gi|15242210 | VDAC3 | 1 |
|  | gi|15242146 | VDAC4 | 6 |
| *Arthroderma gypseum CBS 118893* | gi|315056847 |  | 3 |
| *Arthroderma otae CBS 113480* | gi|296827738 |  | 3 |
| *Aspergillus nidulans FGSC A4* | gi|67528408 |  | 4 |
| *Aspergillus oryzae RIB40* | gi|317146254 |  | 4 |
| *Bos taurus* | gi|73853754 | VDAC1 | 4 |
|  | gi|62177148 | VDAC2 | 1 |
|  | gi|27807415 | VDAC3 | 7 |
| *Botryotinia fuckeliana B05.10* | gi|154316512 |  | 4 |
| *Brassica napus* | gi|56693621 | VDAC1 | 3 |
| *Brassica rapa* | gi|42601787 |  | 3 |
| *Brassica rapa subsp. pekinensis* | gi|44894747 |  | 3 |
| *Caligus rogercresseyi* | gi|225710984 |  | 5 |
| *Camponotus floridanus* | gi|307172337 |  | 1 |
| *Candida dubliniensis CD36* | gi|241948667 |  | 2 |
| *Chaetomium globosum CBS 148.51* | gi|116197779 |  | 4 |
| *Coccidioides immitis RS* | gi|119188149 |  | 5 |
| *Crassostrea gigas* | gi|148717311 |  | 2 |
| *Culex quinquefasciatus* | gi|170029514 |  | 3 |
| *Danio rerio* | gi|47777306 | VDAC1 | -2 |
|  | gi|41054601 | VDAC2 | 4 |
|  | gi|47086069 | VDAC3 | 4 |
| *Dicentrarchus labrax* | gi|317419468 | VDAC2 | 3 |
| *Drosophila melanogaster* | gi|17136632 |  | -1 |
| *Eriocheir sinensis* | gi|307352167 |  | 4 |
| *Gallus gallus* | gi|76443696 | VDAC1 | 0 |
|  | gi\|46048903 | VDAC2 | 3 |
| *Glossina morsitans morsitans* | gi|289743425 |  | -2 |
| *Graphocephala atropunctata* | gi|90820036 |  | 2 |
| *Harpegnathos saltator* | gi|307198439 |  | 2 |
| *Helicoverpa armigera* | gi|328670887 |  | 0 |
| *Homalodisca vitripennis* | gi|46561750 |  | 2 |
| *Homo sapiens* | gi|4507879 | VDAC1 | 3 |
|  | gi|42476281 | VDAC2 | 1 |
|  | gi|208879465 | VDAC3-1 | 6 |
|  | gi|25188179 | VDAC3-2 | 6 |
| *Ictalurus furcatus* | gi|308321947 | VDAC2 | 3 |
| *Ictalurus punctatus* | gi|317575726 | VDAC2 | 4 |
| *Ixodes scapularis* | gi|241061134 |  | 4 |
| *Lodderomyces elongisporus NRRL YB-4239* | gi|149244644 |  | 3 |
| *Lotus japonicus* | gi|36957170 | VDAC1.1 | 2 |
|  | gi|36957183 | VDAC1.2 | 2 |
|  | gi|36957198 | VDAC1.3 | -1 |
|  | gi|36957216 | VDAC2.1 | 5 |
|  | gi|36957233 | VDAC3.1 | 6 |
| *Magnaporthe oryzae 70-15* | gi|145610687 |  | 6 |
| *Mesembryanthemum crystallinum* | gi|1724100 |  | 3 |
| *Metarhizium acridum CQMa 102* | gi|322701176 |  | 3 |
| *Mus musculus* | gi|6755963 | VDAC1 | 3 |
|  | gi|6755965 | VDAC2 | 1 |
|  | gi|312222784 | VDAC3-1 | 7 |
|  | gi|6755967 | VDAC3-2 | 7 |
| *Musca autumnalis* | gi|315259612 |  | 1 |
| *Neosartorya fischeri NRRL 181* | gi|119501158 |  | 4 |
| *Neurospora crassa OR74A* | gi|85100389 |  | 4 |
| *Nicotiana tabacum* | gi|161788872 | VDAC1 | 1 |
|  | gi|161788874 | VDAC2 | 1 |
|  | gi|161788876 | VDAC3 | 2 |
| *Oryctolagus cuniculus* | gi|126723018 | VDAC1 | 3 |
|  | gi|130490906 | VDAC2 | 1 |
|  | gi|126722707 | VDAC3 | 7 |
| *Oryza sativa Japonica Group* | gi|115478779 |  | 0 |
|  | gi|115438000 |  | 0 |
|  | gi|115465125 |  | 2 |
| *Osmerus mordax* | gi|225706314 | VDAC2 | 4 |
| *Ovis aries* | gi|187607533 | VDAC1 | 3 |
| *Paracoccidioides brasiliensis Pb01* | gi|295659414 |  | 4 |
| *Paralichthys olivaceus* | gi|111146880 |  | 3 |
| *Pediculus humanus corporis* | gi|242019706 |  | -2 |
| *Penicillium marneffei ATCC 18224* | gi|212528518 |  | 4 |
| *Pennisetum glaucum* | gi|31249532 |  | 7 |
| *Phaseolus coccineus* | gi|67848430 | VDAC32 | 2 |
| *Pichia angusta DL-1* | gi|320583552 |  | 3 |
| *Pichia pastoris GS115* | gi|254569372 |  | 5 |
| *Pisum sativum* | gi|1172558 | VDAC1 | 5 |
| *Pongo abelii* | gi|197099134 | VDAC3 | 7 |
| *Populus trichocarpa* | gi|224125966 |  | 4 |
|  | gi|224081865 |  | 3 |
|  | gi|224071317 |  | 3 |
|  | gi|224129860 |  | 2 |
|  | gi|224102515 |  | 3 |
|  | gi|224102711 |  | 1 |
|  | gi|224141349 |  | 2 |
| *Prunus armeniaca* | gi|5031279 |  | 0 |
| *Rattus norvegicus* | gi|13786200 | VDAC1 | 3 |
|  | gi|13786202 | VDAC2 | 1 |
|  | gi|13786204 | VDAC3 | 7 |
| *Rhipicephalus microplus* | gi|315142889 |  | 5 |
| *Ricinus communis* | gi|255568593 |  | 5 |
|  | gi|255538066 |  | 3 |
|  | gi|255558216 |  | 1 |
| *Saccharomyces cerevisiae* | gi|173166 |  | 0 |
| *Saccharomyces cerevisiae FostersB* | gi|323304529 | VDAC2 | 10 |
| *Saccharomyces cerevisiae JAY291* | gi|256273731 | VDAC2 | 10 |
| *Saccharomyces cerevisiae RM11-1a* | gi|190406325 |  | 10 |
| *Saccharomyces cerevisiae S288c* | gi|6324273 | VDAC1b | 1 |
| *Saccharomyces cerevisiae YJM789* | gi|151944477 |  | 2 |
| *Saccharomyces cerevisiae YJM789* | gi|151943053 |  | 10 |
| *Salmo salar* | gi|209155200 | VDAC1 | -3 |
|  | gi|197632611 | VDAC2-1 | 3 |
|  | gi|197632615 | VDAC2-3 | 5 |
|  | gi|213511881 | VDAC3 | 5 |
| *Scheffersomyces stipitis CBS 605* | gi|126137904 |  | 2 |
| *Schizosaccharomyces japonicus yFS275* | gi|213401153 |  | 2 |
| *Schizosaccharomyces pombe 972h-* | gi|19115573 |  | 0 |
| *Solanum tuberosum* | gi|1172555 | VDAC34 | 3 |
|  | gi|1172556 | VDAC36 | 1 |
| *Spinacia oleracea* | gi|1256259 |  | -1 |
| *Squalus acanthias* | gi|7637908 |  | 2 |
| *Sus scrofa* | gi|47522750 | VDAC1 | 3 |
|  | gi|47523794 | VDAC2 | 1 |
|  | gi|47523786 | VDAC3 | 6 |
| *Taeniopygia guttata* | gi|197129399 | VDAC3 | 4 |
| *Talaromyces stipitatus ATCC 10500* | gi|242766227 |  | 4 |
| *Triticum aestivum* | gi|1172553 | VDAC1 | 2 |
|  | gi|558650 | VDAC2 | 6 |
|  | gi|558652 | VDAC3 | -1 |
| *Uncinocarpus reesii 1704* | gi|258568258 |  | 3 |
| *Xenopus (Silurana) tropicalis* | gi|62858159 | VDAC1 | 0 |
|  | gi|62859123 | VDAC2 | 3 |
|  | gi|58332202 | VDAC3 | 5 |
| *Xenopus laevis* | gi|147903881 | VDAC1 | 0 |
|  | gi|148225606 | VDAC2 | 2 |
|  | gi|148225606 | VDAC3 | 2 |
| *Zea mays* | gi|162460904 | VDAC1a | 1 |
|  | gi|162459613 | VDAC1b | 1 |
|  | gi|162459730 | VDAC2 | 1 |
