# Supplementary material for: Concentration Dependent Ion Selectivity in VDAC: A Molecular Dynamics Simulation Study
Source: PLoS One. 2011 Dec 2;6(12):e27994. doi: 10.1371/journal.pone.0027994 (PMC3229507; doi:10.1371/journal.pone.0027994)
Supplement: Table S2 — Radial distribution properties computed from simulations of 0.1 M and 1.0 M KCl solutions. The distances for the first and second peak (rmax) and minima (rmin), the height of the peak g(rmax) and of the minima g(rmin), as well as the integration number of the RDF up to its first (second) minimum (N(rmin)) are listed. The RDF of potassium-chloride interaction at 0.1 M and 1.0 M show qualitatively the same behavior. However, at 0.1 M KCl the accurate determination of the potassium-chloride interaction parameters was prevented due to large fluctuations in the RDF (see Figure S8). All distances are given in Å. (DOC) [file pone.0027994.s010.doc]

| Pairs | Peak/ Minima | r_max_ | g(r_max_) | r_min_ | g(r_min_) | **N(r_min_)** |
| --- | --- | --- | --- | --- | --- | --- |
| K^+^-Cl^-^ | first | -/3.00 | -/14.65 | -3.95/ | -/0.28 | -/0.39 |
| (0.1 M/1.0 M) | second | -/5.40 | -/2.00 | -/6.37 | -/0.89 | -/1.17 |
| K^+^-O | first | 2.71/2.70 | 8.77/4.82 | 3.57/3.54 | 0.66/0.41 | 6.73/6.33 |
| (0.1 M/1.0 M) | second | 4.96/4.90 | 2.1/1.18 | 6.07/6.03 | 1.52/0.94 | 30.03/30.18 |
| Cl^-^-O | first | 3.13/3.14 | 7.63/4.23 | 3.76/3.76 | 0.86/0.58 | 7.26/7.23 |
| (0.1 M/1.0 M) | second | 5.21/5.29 | 1.86/1.06 | 6.29/6.29 | 1.53/0.94 | 32.30/33.52 |
